# Health Tract No. 324

**Published:** 1868-07

**Authors:** 


HEALTH TRACT No. 324.

### STOMACH COUGH.

Sometimes the throat tickles and excites cough or is otherwise sore or ailing, tickling is felt in the throat because the stomach has not been able to digest the food which has been taken into it; when that is the case the food ferments, sours, generates wind, a sour gas, or fluid, sometimes so acrid as to take the skin off the tongue and lips in its outward passage, but having to come along through the throat its tender surface is also affected, but not so decidedly, because the parts are not so sensitive, are not so largely supplied with nerves, hence it requires frequent indigestions, souring of the food, and in process of time the throat does begin to complain; it becomes inflamed, manifesting itself by a pricking sensation, a dryness, a hurting, an annoying and fruitless swallowing or actual cough and ulceration; but it is utterly useless to cure this by applying any thing to the throat, as long as the condition of the stomach is unchanged; but when that is rectified, when the cause of the throat ail is removed, that is when the indigestion, when the dyspeptia is cured; the throat will get well of itself. Hence when a man has a cough, arising from a tickling sensation in the throat, the most important question is, does that tickling arise from the condition of the stomach, or the lungs and then to address the remedies to whichever of these points gives rise to the tickling.

Not all who are dyspeptic have trouble in the throat, because the dyspeptia, the indigestion is not always in the stomach, it may be lower down in the alimentary canal and gases generated there do not pass to the throat, but in another direction.

It may be safely said that four cases out of five where there is some trouble in the throat without any actual cough, it is of dyspeptic origin, is in connection with improper eating or a disordered liver and to expect to cure the case by applications to the throat, when the disease is, really at a point near two feet distant is irrational and any such applications must fail.

